# Commencement of Lecture Terms in Medical Colleges, &c.

**Published:** 1871-10

**Authors:** 


### Editorial.

### Commencement of Lecture Terms in Medical Colleges, &c.

It may be well at the present time, when the Lecture term in our own, and in most of the Medical Colleges of the country, is about to commence, to draw attention to the general fact, and speak a word of the advantages thus offered Medical students to obtain knowledge of the scienee and art of medicine. It is not very uncommon to hear our colleges spoken of, as close corporations, organized for secondary and unworthy objects, superficial in their teaching, and requiring but little attainment for graduation. Such statements have not been made, perhaps, without some show of truth in rare instances, where new colleges were struggling for a beginning; but so far as obtains in the vast majority of Medical Colleges in our country, nothing can be farther from the truth. It will no doubt be conceded by all, that in our American Colleges have been educated our American physicians, and cannot these institutions point to the physicians of the country with pride, and say, "*ye are our witnesses.*" If they have borne such fruit, is there just ground to believe them inferior in their modes of instruction, or inadequate in the scope and extent of their teachings. Prof. Gross, writing upon the comparative standing and attainments of American physicians, says, " If we compare the results of the practice and operations of American with those of European Surgeons, it will be found that they are fully up to the general average, if, indeed, not far in advance. This statement is true alike of our civil and military experience. Where are there better, more enlightened, more able, skillful, or scientific staffs than are to be daily seen in the wards of the hospitals of our cities? Comparisons are odious. In my visits last summer to the hospitals of France, Italy, Austria, Prussia, Holland, Belgium, England, Scotland and Ireland, I saw nothing to induce the belief that the Surgeons of these institutions, many of them veterans, whose names are as familiar to the American Medical profession as household words, are, in any respect superior to the same class of men among us, either in general or professional intelligence, in the art of diagnosis, in therapentic refinement, or in manual dexterity."

When Chassainac affirms, " America at this moment wields the Surgical sceptre of the world, " it can scarcely be made to mean that American Surgeons are destitute of education, skill and scientific attainments. It is expected of our Medical Colleges that they educate young men as far as possible in medicine, surgery and the collateral sciences—Anatomy, Physiology, Chemistry, etc., etc. How well and faithfully this has been, and is being done, is sufficiently obvious. Whatever might be said of the requirements of foreign institutions in comparison with our own—of the length of time and preliminary culture demanded, whatever is claimed for their graduates in being more learned—knowing more Greek and Latin, Mathematics, Astronomy, and other branches of university education, no one will suppose them more thoroughly trained in a full knowledge of Medicine, Surgery, and the collateral sciences. American physicians educated and graduated from American institutions have superiority in practical knowledge, which has been everywhere seen, and frequently acknowledged, and is just source of honest pride. The reason of this superiority in practical wisdom we do not propose at present to explain, they are sufficiently obvious. It is not claimed that our Medical Colleges have reached the highest point of possible excellence, or that the standard of preliminary and professional attainment is as high as is desirable, far from it. It is only claimed that the American Medical student may here obtain as perfect knowledge of medicine and the collateral sciences as can be obtained in the same time in any college or country. We have defects in our system of education, and we shall be with the very first in advocating any changes for improvement. Meanwhile we most heartily congratulate the medical students of to-day upon the exalted and ample opportunities which they may enjoy. No country or profession ever made more rapid strides in improvement than has been witnessed in our conntry and by the American medical profession, and we have just ground of pride in our achievements. If it is an honor to be an American citizen, by how much greater honor to be an American physician.

